# Ticks (Acari: Ixodidae) and tick-borne agents associated with domestic dogs in an environmental protection area in Brazil, with molecular evidence of *Rhipicephalus linnaei* (Audouin, 1826)

**DOI:** 10.1590/S1984-29612024045

**Published:** 2024-10-07

**Authors:** Hélio Freitas Santos, Walter Flausino, Thiago Fernandes Martins, Igor Silva Silito, Hermes Ribeiro Luz, Maria Carolina de Azevedo Serpa, Marcelo Bahia Labruna, João Luiz Horacio Faccini

**Affiliations:** 1 Departamento de Parasitologia Animal, Universidade Federal Rural do Rio de Janeiro – UFRRJ, Seropérica, RJ, Brasil; 2 Departamento de Medicina Veterinária Preventina e Saúde Animal, Faculdade de Medicina Veterinária e Ciência Animal, Universidade de São Paulo – USP, São Paulo, SP, Brasil; 3 Programa de Pós-graduação em Ciências da Saúde, Centro de Ciências Biológicas e da Saúde, Rede Nordeste de Biotecnologia – RENORBIO, Universidade Federal do Maranhão – UFMA, São Luís, MA, Brasil

**Keywords:** Dogs, wildlife, Ixodidae, Atlantic forest, Rickettsia bellii, Cães, animais silvestres, Ixodidae, Floresta Atlântica, Rickettsia bellii

## Abstract

Wild animals and domestic dogs living in human dwellings near forested areas can share ectoparasites, including ticks. In this study, we surveyed ticks associated with dogs which tutors living in the Palmares Environmental Protection Area (EPA Palmares). Dogs were classified into three categories, domiciled, semi-domiciled and wandering dogs according to dog care/ type of dwelling. Ticks were collected monthly from January to December, 2020. Overall, 60 (33.9%) out of 177 examined dogs were infested by ticks. Six species of ticks were identified: *Rhipicephalus linnaei*, *Amblyomma aureolatum, Amblyomma sculptum, Amblyomma ovale, Amblyomma dubitatum* and *Rhipicephalus microplus*. The overall prevalence and presence in semi-domicilied+wandering dogs was higher for *A. aureolatum* than for *R. linnaei* by the Chi-square statistic tests. A random sample of 50 ticks, collected from 22 different dogs, were processed through molecular analyses. Ticks were submitted to DNA extraction and also by PCR, using specific primers in order to pathogens monitoring. Four males of *A. aureolatum* yielded DNA sequences (350 bp) that were 100% identical to the type strain of *Rickettsia bellii* in GenBank (CP000087).

## Resumo

Animais silvestres e cães domésticos que vivem em habitações humanas, próximas a áreas florestais, podem compartilhar ectoparasitas, incluindo os carrapatos. Neste estudo, foi realizada uma pesquisa sobre carrapatos, associados a cães de tutores residentes na Área de Proteção Ambiental Palmares (APA Palmares). Os cães foram classificados em três categorias; domiciliados, semidomiciliados e errantes, de acordo com os cuidados do cão e tipo de moradia. As coletas de carrapatos foram realizadas, mensalmente, de janeiro a dezembro de 2020. No total, 60 (33,9%) dos 177 cães examinados estavam infestados por carrapatos. Foram identificadas seis espécies: *Rhipicephalus linnaei, Amblyomma aureolatum, Amblyomma sculptum, Amblyomma ovale, Amblyomma dubitatum* e *Rhipicephalus microplus.* A prevalência geral e a presença em cães semidomiciliados + cães errantes foi mais elevada para *A. aureolatum* do que para *R. linnaei*, de acordo com os resultados do testes do qui-quadrado. Uma amostra aleatória de 50 carrapatos, coletados de 22 cães diferentes, foi processada por meio de análises moleculares. Os carrapatos foram submetidos à extração de DNA e também por PCR, utilizando-sw primers específicos para monitoramento de patógenos. Quatro machos de *A. aureolatum* produziram sequências de DNA (350 bp) que eram 100% idênticas à cepa tipo de *Rickettsia bellii* no GenBank (CP000087).

## Introduction

The relationship between ticks, domestic dogs and humans in rural areas is a matter of growing veterinary and public health concern worldwide because of the increasing contact between domestic dogs and wildlife due to deforestation for anthropic activities (Bodo et al., 2021). Domestic dogs that enter forested areas are likely to become infested by a wide spectrum of ticks primarily associated with wildlife. In turn, these ticks can transmit pathogens to domestic dogs and humans (as zoonoses) worldwide (Saleh et al., 2021); and vice versa, infested dogs might introduce ticks and pathogenic agents (e.g. *Rickettsia* sp., *Babesia* sp., *Ehrlichia* sp. and *Anaplasma* sp.) to wildlife.

In South America, the taxon *Rhipicephalus sanguineus* sensu lato (s.l.) is represented by two distinct species. One of this species is *R. sanguineus* sensu stricto (s.s.), formerly called the “temperate species”, which is currently established in the southern cone of the continent, i.e. Argentina, Chile, Uruguay and southern Brazil (Moraes-Filho et al., 2011, Nava et al., 2018). The other species is the *R. sanguineus* “tropical species”, which is possibly present in all tropical and subtropical regions of South America (Burlini et al., 2010, Moraes-Filho et al., 2011). In a recent study, the species *Rhipicephalus linnaei* was resurrected to represent the so-called “tropical species” of *R. sanguineus* s.l. (Šlapeta et al., 2021, 2022).

In Brazil, domestic dogs can become infested by ticks in two scenarios: (i) in urban areas, where they are confined to a limited space and without contact with forested environments; and (ii) in rural areas with or without access to forested areas. In the first scenario, the most common ticks are *R. sanguineus* s.l. In the second one, besides the aforementioned species, the tick species currently most commonly associated with domestic dogs are *Amblyomma aureolatum, Amblyomma ovale, Amblyomma sculptum* and/or *Amblyomma tigrinum* (Labruna & Pereira., 2001; Luz et al., 2014). Information on ticks and tick-borne pathogens of domestic dogs in this scenario is still limited in comparison with urban areas and is primarily focused on tick-borne pathogen surveys, whereas the relationship between domestic dog-tick parasitism is usually dealt with superficially (O'Dwyer et al., 2001; Sousa et al., 2017; Paulino et al., 2018).

*Rhipicephalus linnaei* (cited as *R. sanguineus*) ticks are proven vectors of three canine pathogens: *Babesia vogeli*, the etiological agent of canine babesiosis; *Ehrlichia canis*, the etiological agent of canine monocytic ehrlichiosis (Labruna & Pereira, 2001); and *Rickettsia rickettsii,* the etiological agent of Brazilian spotted fever (Labruna et al., 2009; Piranda et al., 2008, 2011).

*Amblyomma aureolatum* is a proven vector of the piroplasmid *Rangelia vitalli* to domestic dogs (Soares et al., 2018) and of the bacterium *R. rickettsii* to dogs and humans in the São Paulo City Metropolitan area (Pinter & Labruna, 2006). This tick, an autochthonous species widely distributed throughout Brazil, is primarily associated with wildlife. Adults feed on wild carnivores, whereas immature forms feed chiefly on passerine birds and occasionally on small mammals (Ogrzewalska & Pinter, 2016). Its natural habitat in Brazil encompasses high altitudes of the Atlantic Forest of southeastern and southern Brazil, although it is also found at low altitudes in southern Brazil (Barbieri et al., 2015; Faccini et al., 2022).

*Amblyomma ovale* is a proven vector of *Hepatozoon canis* to domestic dogs (Forlano et al., 2005; Rubini et al., 2009). This species has also been associated with cases of milder spotted fever caused by the Atlantic rainforest strain of *Rickettsia parkeri* (Sevá et al., 2019). This is another autochthonous species that is widely distributed throughout Brazil (Labruna et al., 2005), but *A. ovale* is usually absent from high altitudes of the Atlantic Forest of southeastern and southern Brazil (Barbieri et al., 2015). *Amblyomma ovale* is primarily associated with wildlife, like *A. aureolatum*. Adults feed on wild carnivores whereas immature forms feed chiefly on small mammals, and occasionally on birds (Ogrzewalska & Pinter, 2016).

*Amblyomma sculptum* is the main vector of *R. rickettsii* to humans in Brazil, and to some extent to domestic animals, and may play a role in the transmission of *Theileria equi* to horses (Paula et al., 2022). This species is distributed in Brazil, Bolivia, Paraguay and northern Argentina. Its distribution in Brazil generally encompasses the entire Cerrado and Pantanal biomes and a large part of the Atlantic forest biome (Martins et al., 2016).

This study was primarily set up to investigate the association between ixodid ticks and domestic dogs that had free access into forests in an environmental protection area (APA) located in the state of Rio de Janeiro, southeastern Brazil, with possible detection of *Rickettsia* and *Borrelia* in ticks.

## Material and Methods

### Study site

The APA Palmares is located in the southern region of the municipality of Paty do Alferes, between the coordinates 22º26' - 22º28' S and 43º22' - 43º26' W. It occupies an area of 1,485.5 ha and is part of the vegetation zone of dense montane ombrophilous forest, with altitudes ranging from 676 to 1216 m. The climate is close to that proposed by Köppen as tropical with periodic rain and dry winter (Aw) (Paiva & Lopes, 2013).

### Tick collection and morphological identification

Tick collections were carried out at altitudes ranging from 829 to 998 m above sea level. Dogs were grouped into three categories as proposed by Reichmann et al. (2000): dogs supervised or controlled by an owner, family dogs and wandering dogs, henceforward named, respectively, resident dogs, semi-resident dogs and wandering dogs. The resident dogs did not have access to the common area of the APA Palmares and were kept inside walled or screen-fenced residences. The semi-resident dogs, despite having a keeper, had free access to the entire area of the APA Palmares. The wandering dogs did not have any keeper and habitually roamed freely in the APA, using abandoned structures as shelters and occasionally receiving human food waste from residents.

A convenience sample of 177 mongrel dogs was surveyed from January to December 2020, and each animal was examined for a period of approximately five minutes. Ticks were collected with the help of tweezers during a detailed inspection of the following body regions: head, ears, neck, chest, abdomen, fore and hind limbs, interdigital areas, axillae, tail and inguinal regions. Through this visual inspection, all detectable ticks (adults and nymphs) on each dog were collected. The adult ticks collected were morphologically identified as described by Dantas-Torres et al. (2019) and Šlapeta et al. (2022), using a stereoscope microscope.

### Molecular analyses

A random sample of 50 ticks, collected from 22 different dogs, were processed through molecular analyses. These ticks consisted of 38 *A. aureolatum* (28 males, 8 females, 2 nymphs)*,* 11 *R. linnaei* (6 males, 5 females) and one *A. ovale* female. Each tick was individually subjected to DNA extraction using the guanidine isothiocyanate phenol technique (Sangioni et al., 2005) and tested through a polymerase chain reaction (PCR) assay targeting a fragment of approximately 460 bp of the tick 16S rRNA mitochondrial gene, as described by Mangold et al. (1998). PCR products were electrophoresed in 1.5% agarose gel to confirm DNA amplification and successful DNA extraction.

Thereafter, the 50 tick DNA samples were tested using two protocols of Taqman real-time PCR assays. The first one targeted the citrate synthase gene (*gltA*) of bacteria of the genus *Rickettsia*, as previously described (Labruna et al., 2004; Soares et al., 2015). Samples found to be positive through this Taqman real-time PCR (cycle threshold ≤ 35) were tested using three protocols of conventional PCR: one targeting an 401 bp fragment of the *gltA* gene of members of the genus *Rickettsia* (Labruna et al., 2004); one species-specific assay targeting a 338 bp fragment of *Rickettsia bellii* (Szabó et al., 2013); and one targeting a fragment of approximately 630 bp of the rickettsial 190-kDa outer membrane protein gene (*ompA*) of *Rickettsia* spp. of the spotted fever group, as described by Eremeeva et al. (2006). In each set of reactions, negative control tubes containing water and a positive control tube containing *Rickettsia vini* DNA were included.

The second protocol of Taqman real-time PCR assay targeted the 16S rRNA gene of organisms of the genus *Borrelia*, as described by Parola et al. (2011). Negative control tubes containing water and a positive control tube containing *Borrelia anserina* DNA were included. PCR products of the conventional PCR assay for the rickettsial *gltA* gene were purified and sequenced with the Big Dye Terminator cycle sequencing kit (Applied Biosystems, Foster City, CA, USA) in an automatic sequencer (model ABI 3500 Genetic Analyzer; Applied Biosystems) in accordance with the manufacturer’s protocol. The sequences generated were subjected to BLAST analysis (Altschul et al., 1990) to infer the closest similarities to tick DNA sequences available in GenBank. In addition, we also generated partial sequences for the mitochondrial 16S rRNA gene of eight *R. linnaei* ticks, in order to confirm their taxonomic identities.

### Statistical analyses

We used only adults of *R. linnaei* and *A. aureolatum* because only these stages of these species had statically significant sample sizes, respectively, 143 and 119 adults. Although some dogs were resampled for tick collection at intervals ranging from one to five months, each dog examined in a given month during the survey was taken to be an independent unit. We assumed that monthly tick collections belonged to different cohorts. The rationale was that all adult ticks and nymphs were collected each month and that the larval parasitic periods of *R. linnaei* (Bechara et al., 1995), *A. aureolatum* (Rodrigues et al., 2002) and *A. sculptum* (Prata et al., 1997), respectively, were 5 ± 0.6 days (as *R. sanguineus*), 3 to 8 days and 4 to 6 days. We used the chi-square statistical test (Dean et al., 2010) to estimate possible differences in overall prevalence regarding associations between adults of *A. aureolatum* and *R. linnaei* and the category of semi-resident dogs + wandering dogs, and associations with dog sex. These two categories were summed because of the low number of wandering dogs surveyed and because all dogs in these two categories were likely to have similar infestations given that they had free access to forested areas. We focused only on associations of adults of *R. linnaei* and *A. aureolatum* because adequately sized samples of these two species were obtained, in contrast to the small sample sizes of the remaining tick species found in this study*.* Values of p < 0.05 (two-tailed) were considered statistically significant.

Voucher specimens were deposited in the tick collection “Coleção Nacional de Carrapatos Danilo Gonçalves Saraiva” (São Paulo, SP, Brazil) under accession numbers CNC 4709 to 4711.

## Results

### Ticks on dogs

We examined 177 mongrel dogs in the APA Palmares from January to December 2020. Overall, 65% (115/177) of the dogs were classified as semi-resident dogs, 31.1% (55/177) as resident dogs and 4% (7/177) as wandering dogs.

A total of 352 tick specimens were collected and identified into six different species. The most abundant species were *R. linnaei* (85♂ + 58♀ + 24NN = 167) and *Amblyomma aureolatum* (46♂ + 73♀ + 1N = 120). A further four tick species were collected but not included in the analysis of tick-dog-relationships due to small sample size: *Amblyomma sculptum* (1♂ + 1♀ + 54NN = 56) on 34 dogs; *Amblyomma ovale* (1♂ + 2♀ = 3) on three dogs; *Rhipicephalus microplus* (4♀ + 1N = 5) on three dogs, and *Amblyomma dubitatum* (1N) on one dog. Thus, the data on adults of *R. linnaei* and *A. aureolatum,* shown in Tables 1 and 2, apply to only 59/177 (33.9%) infested dogs. The overall prevalence (Table 1) and presence among semi-resident + wandering dogs (Table 2) was higher for *A. aureolatum* than for *R. linnaei*, according to the chi-square statistical test for overall prevalence (Table 1) [χ^2^ (1, N = 59) = 6.72, p = 0.09534 (two-tail), p < 0.05] and for presence among semi-resident + wandering dogs (Table 2) [χ^2^ (1, N = 115) = 3.026, p = 0.08194 (two-tail), p < 0.05].

**Table 1:** Overall abundance (AB), prevalence (PR) and mean intensity of infestation (MI) of adults of *Rhipicephalus linnaei* and *Amblyomma aureolatum* collected on 59 dogs out of 177 examined in the APA Palmares, from January to December, 2020. The AB was assessed individually only for *R. linnaei* and *A. aureolatum*.

Ticks	Dogs	AB (%)	PR (%)	MI
*R. linnaei*	18	143 (54.6)	10.2	143/18=7.9
*A. aureolatum*	32	119 (45.4)	18.1	119/32=3.7
*A. aureolatum* + *R. linnaei*	9	--	5.1	---
**Total**	59	262	33.3	

**Table 2:** Prevalence of adults of *Rhipicephalus linnaei* and *Amblyomma aureolatum* collected on 59 dogs of the group dog care/residence in the APA Palmares, RJ, from January to December, 2020.

Dogs	Dog care/Residence			
	Resident	Semi-resident	Wandering	Total
Dogs examined	55 (31.1%)	115 (65.0%)	7 (4%)	177
Dogs infested	10 (18.2%)	45 (39.1%)	4 (57.1%)	59 (33.3%)
*R. linnaei*	3 (30%)	14 (31.1%)	1 (25%)	18 (30.5%)
*A. aureolatum*	7 (70%)	24 (53.3%)	1 (25%)	32 (54.2%)
*A. aureolatum* + *R. linnaei*	0	7 (15.6%)	2 (50%)	9 (15.3%)

We detected infestations by *R. linnaei* and/or *A. aureolatum* throughout the study period (January to December 2020). Re-infestations were seen on 38.3% (23/60) of the dogs, distributed as follows: 47.8% (11/23) of the dogs re-infested only with *A. aureolatum*, 13% (3/23) of the dogs re-infested only with *R. linnaei* and 39.1% (9/23) of the dogs re-infested with both species. Although the infestations on the different parts of the dog bodies were not evaluated numerically, we observed that the main parts parasitized by *R. linnaei* adults were the neck, back, interdigital space, ear and paw; while for *A. aureolatum* adults, the main parts were the neck, back, ear, around the eyes and cheek.

The following procedure was used to calculate the chi-square results for the possible dependence between tick species and host sex: in mixed infestations, each tick species was counted individually including ticks in concomitant infestations. Thus, the total number of parasitized dogs (Table 3) exceeds the total of 59 infested dogs. Although most of the ticks of both species were seen on male dogs (Table 3), there was a non-significant statistical difference, [χ^2^ (1, N = 69) = 0.1234, p = 0.7253 (two-tail), p > 0.05].

**Table 3:** Number of adult ticks collected according to dog sex in the APA Palmares, from to January to December, 2020.

Ticks	Dogs		
	Males	Females	Total
*R. linnaei*	18	8	26
*A. aureolatum*	28	15	43
Total	46	23	69

**Figure 1:**
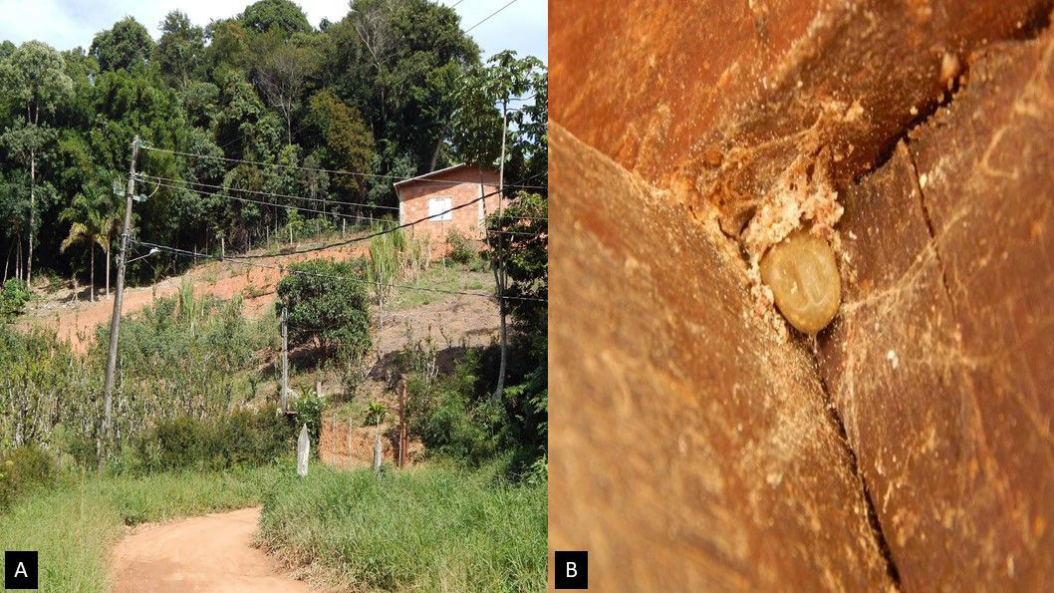
Close-up of a residence located nearby the forested area of the APA Palmares and an engorged female of *Rhipicephalus linnaei* ovipositing at 2.07m high in a house. (A) location where the female was collected; (B) female of *R. linnaei.*

In one house, we recorded upward movement of 12 engorged females of *R. linnaei* and oviposition in crevices of a pillar in a garage, at heights above the floor from 62 cm to 2.07 m (Figure 1).

### Molecular analyses

The results from the molecular analyses on all the tick samples yielded amplicons of the expected size through PCR targeting the mitochondrial 16S rRNA gene, thus indicating the presence of viable DNA. Through the two protocols of Taqman real-time PCR assays, no borrelial DNA was amplified, whereas rickettsial DNA was amplified from four *A. aureolatum* males. These four ticks also yielded amplicons through the conventional *gltA-*PCR assay and the *R. bellii-*specific PCR assay. Amplicons from the later assay generated DNA sequences 100% identical to the type strain of *R. bellii* in GenBank (CP000087). No amplicon was generated for the rickettsial *ompA-*PCR assay. Partial sequences for the mitochondrial 16S rRNA gene of four *R. linnaei* ticks (three males and one female from different dogs) were 100% identical to sequences of *R. linnaei* from GenBank (MW429382, MW429383). The haplotypes of *R. bellii* (*gltA* gene) and *R. linnaei* (16S rRNA gene) generated in this study have been submitted to GenBank under accession numbers OR961479 and OR797057, respectively.

## Discussion

### Ticks on dogs

The finding of *R. linnaei* and *Amblyomma* spp. as the most prevalent tick species in this study is in agreement with previous publications using data from rural areas in Brazil (Labruna et al., 2000; Dantas-Torres et al., 2009; Costa et al., 2013). However, differences in tick-dog relationships, compared with our results, are to be expected because of the ecological differences of the regions where those previous studies were carried out. Our results indicated that adults of both *R. linnaei* and *A. aureolatum* are present throughout the year, although it was not possible to establish a seasonal pattern of infestation because the present survey was not designed for this purpose. These results in Neotropical southeastern Brazil were rather expected and were similar to the findings of Pinter et al. (2004), Labruna & Faccini (2020) and Paula et al. (2022), who collected adults of *A. aureolatum, Dermacentor nitens* and *Amblyomma sculptum,* respectively, throughout the year.

The re-infestations of some dogs by either *R. linnaei* or *A. aureolatum*, or by both species, suggest that these mongrel dogs were susceptible to (re-)infestations by both species. Regarding *R. linnaei*, the re-infestations seen in this survey were in line with what was observed by Szabó et al. (1995), who experimentally re-infested mixed-breed dogs with this tick. However, this issue still requires further investigation, as Inokuma et al. (1997) demonstrated that there was some resistance to *R. linnaei* infestations among Beagle dogs, and Louly et al. (2009) showed this in relation to both Beagles and English Cocker Spaniels. Re-infestations by *A. aureolatum* could also be the result of host susceptibility to this tick, which was previously shown under natural conditions (Pinter et al., 2004).

Sex-biased parasitism has been observed in numerous mammal species. However, the mechanisms behind this pattern are still debatable because parasite populations are affected by a number of factors, such as host population density, habitat selection, reproductive strategies and behavioral patterns, which can interact with host sex. We can argue that the behavior of dogs inhabiting environmental protected areas in Brazil corresponds in some way to that of wild mammals. Therefore, we have not extrapolated the results regarding the association between tick infestation and the sex of dogs living in cities, as done by Silveira et al. (2009) and Lourenço et al. (2020).

The larger proportion of male ticks, compared with female ticks, seen in relation to both tick species, may have been due to the fact that females detach from the host for oviposition, while males remain attached for a longer period of time, as reported by Pinter et al. (2002) for *Amblyomma sculptum*.

The larger numbers of nymphs of *R. linnaei* and *A. sculptum* were expected because all active stages of both species feed on dogs. In addition, the nidicolous behavior of *R. linnaei* also favors reinfestation because of its intimate associations with dogs in their shelters, where it hides in cracks and crevices. The higher prevalence of *R. linnaei* on semi-domiciled dogs (Table 2), in comparison with domiciled dogs, can be explained by the fact that the former also take shelter under abandoned man-made facilities or farm machinery on farm near forested areas, where these dogs tend to spend a few hours a day (Labruna & Pereira, 2001), as seen in APA Palmares. Also, the same line of reasoning can be applied to the finding of *R. linnaei* in 75% (3/4) of the wandering dogs.

The height above ground level reached by one engorged female (2.07 m), among others, confirms the observation of Labruna & Pereira (2001), who saw females crossing a dividing brick wall between two houses that was approximately 2.5 meters high. Further studies on upward movement of *R. linnaei* are needed in order to clarify tick transmission between dogs and to establish effective control programs for these species. Otherwise, parasitism by *A. sculptum* is rather expected because domestic dogs living in rural areas are frequently seen bearing this tick species (Horta et al., 2007), particularly when horses and dogs share the same space, as seen in the APA Palmares, because it is common for horse owners to also own dogs. In the APA Palmares, some residents use horses for traveling within the APA or between the APA and the municipality of Paty do Alferes, and these animals are kept close to their homes, in small paddocks or tied at the side of roads, where they feed on grass.

The association between adults of *A. aureolatum* and dogs in the study area was also expected because small rodents and birds are hosts for immatures of these ixodids, while wild carnivores and domestic dogs are hosts for adult ticks, as previously mentioned (Ogrzewalska & Pinter, 2016). According to residents, contact between semi-resident dogs and wildlife is rather constant, thus facilitating dispersion/transmission of ticks and tick-borne diseases within two different scenarios. First, it is common for semi-resident dogs to return at dawn with some sort of wildlife that they had hunted during the preceding night in the nearby forested areas of the APA Palmares. Indeed, some dogs are also found to have become injured when they return home after coming into contact with wildlife. Second, residences are regularly visited by some species of wildlife such as opossums (*Didelphis aurita*), dwarf porcupines (*Sphiggurus villosus*), squirrels (*Sciurus aestuans*), wild rabbits (*Silvilagus brasiliensis*), marmosets, also called tamarins (*Callithrix jacchus* and *C. penicillata*), and the Brazilian guinea pig (*Cavia aperea*). In addition, the survey team also saw armadillos (*Dasypus novemcinctus*) and marmosets on the roads and trails across the APA Palmares. Birds such as jacus (*Penelope obscura*) and white-winged doves (*Patagioenas picazuro*) were also seen in nearby backyards, around chicken coops, in search of spilled food. Regarding *A. ovale*, our finding of parasitism by three adults on three dogs should be considered occasional at the moment because its distribution range is typically at lower altitudes, in comparison with the distribution range of *A. aureolatum*, which prefers higher altitudes in the state of Rio de Janeiro (Faccini et al., 2022). Since we did not obtain these dogs’ histories for the days prior to our collection of ticks, we cannot rule out the possibility that they may have acquired *A. ovale* from visiting low-altitude areas around our collection site. For instance, this possibility was confirmed by Sabatini et al. (2010), when collecting *Amblyomma* spp. ticks from dogs at higher altitudes in the state of São Paulo.

Our occasional records of *R. microplus* and *A. dubitatum* on a few dogs should be linked to their sharing of living areas with cattle and capybaras, which are, respectively, the primary hosts for these two tick species (Nava et al., 2017). Under such circumstances, *R. microplus* and *A. dubitatum* have been reported on domestic dogs in Brazil (Labruna et al., 2002; Lourenço et al., 2020). A few head of dairy cattle that were farmed for subsistence, along with two capybaras resting on the shore of a lake, were seen in the APA Palmares.

### Molecular analyses

Recently, Šlapeta et al. (2022) assigned the “tropical species” of *R. sanguineus* s.l. to the taxon *R. linnaei,* which has been confirmed through molecular analyses to occur in many tropical regions of the planet, including Brazil. Subsequently, there were two reports of *R. linnaei* in Brazil, without molecular confirmation (Macedo et al., 2022; Sodelli et al., 2023). In the present study, we identified 167 ticks morphologically as *R. sanguineus* s.l.; however, partial DNA sequences of four specimens of these ticks matched 100% with sequences of *R. linnaei* reported by Šlapeta et al. (2021). Since *R. linnaei* was assigned to the so called “tropical species” of *R. sanguineus* s.l. (Šlapeta et al., 2021, 2022), and because this “tropical species” was reported as the only member of the *R. sanguineus* s.l. species complex to occur in southeastern Brazil (Moraes-Filho et al., 2011), our molecular analyses allow us to state that all *R. sanguineus* s.l. specimens of the present study belong to the species *R. linnaei.*

The detection of *R. bellii* in 10.5% (4/38) of the specimens of *A. aureolatum* in the state of Rio de Janeiro was rather expected, given that Pinter & Labruna (2006) found that the prevalence of *A. aureolatum* infection in the state of São Paulo was 1.5% (10/669), and McIntosh et al. (2015) listed 13 species of *Amblyomma, Ixodes loricatus* and *Haemaphysalis juxtakochi* in which *R. bellii* has been detected in Brazil.

Lastly, this study contributes to enhancing the knowledge about dog-tick relationships in situations in which the keepers of these dogs live in environmental protection areas, a common scenario seen in Brazil.
